# Mouse models of systemic juvenile idiopathic arthritis and macrophage activation syndrome

**DOI:** 10.1186/s13075-023-03032-8

**Published:** 2023-03-25

**Authors:** Natsumi Inoue, Grant S. Schulert

**Affiliations:** 1 Division of Rheumatology, Cincinnati Children’s Hospital Medical Center, Cincinnati, OH USA; 2 Department of Pediatrics, University of Cincinnati College of Medicine, 3333 Burnet Avenue, Cincinnati, OH MLC 4010 USA

**Keywords:** Cytokine storm syndromes, Hemophagocytic lymphohistiocytosis, Still’s spectrum, Animal model, Lung disease

## Abstract

Macrophage activation syndrome (MAS) is a life-threatening complication of pediatric rheumatic diseases, occurring most commonly in children with systemic juvenile idiopathic arthritis (SJIA). Despite several classes of currently available treatment options for SJIA, including biologic agents targeting IL-1 or IL-6, there remain severe cases suffering from refractory disease and recurrent MAS. The phenotype of MAS is similar to hemophagocytic lymphohistiocytosis (HLH), but the underlying pathophysiology of MAS complicating SJIA or other disorders has not been fully clarified. These facts make it challenging to develop and utilize animal models to study MAS. To date, there is no “perfect” model replicating MAS, but several models do demonstrate aspects of SJIA and/or MAS. In this review, we examine the proposed animal models of SJIA and MAS, focusing on how they reflect these disorders, what we have learned from the models, and potential future research questions. As we better understand the key features of each, animal models can be powerful tools to further define the pathophysiology of SJIA and MAS, and develop new treatment targets and strategies.

## Background

Macrophage activation syndrome (MAS) was originally described by rheumatologists as a potentially fatal complication of rheumatic diseases, characterized by systemic inflammatory features such as fever, hepatosplenomegaly, pancytopenia, liver injury, and hypofibrinogenemia with hemophagocytosis in bone marrow [1]. These features resemble the “cytokine storm syndromes,” and have strong similarity to hemophagocytic lymphohistiocytosis (HLH). Historically used by hematologists, HLH is a comprehensive clinical concept and is defined by criteria proposed with the HLH-2004 diagnostic guidelines [2], in which various underlying causes lead to immune dysregulation. HLH is broadly divided into primary HLH (pHLH) with Mendelian inherited conditions, and secondary HLH according to the background pathophysiology. In the most recent histiocytic classification, MAS was defined as a form of secondary HLH occurring in the setting of rheumatic disease, and the term “MAS-HLH” was proposed [3].

MAS can complicate various pediatric rheumatic diseases, but is most commonly seen in patients with systemic juvenile idiopathic arthritis (SJIA) and its adult counterpart adult-onset Still’s disease (AOSD), collectively considered as the Stills spectrum disorders [4–8]. SJIA is a distinct subtype of JIA characterized by systemic inflammatory features such as quotidian fever, evanescent rash, generalized lymphadenopathy, hepatosplenomegaly and serositis along with arthritis [9]. Approximately 10% of patients with SJIA develop clinically overt or “full-blown” MAS [10, 11]. Recently, a high fatality lung disease (LD) complicating SJIA was also reported and has been linked to patients with recurrent MAS episodes [12–14]. While MAS is thought to be a form of secondary HLH, there are some limitations when HLH-2004 criteria are used to diagnose MAS. For example, some thresholds (such as platelet count and fibrinogen level) are not suitable for MAS because of underlying and preceding inflammatory activity of SJIA/AOSD. To overcome this problem, preliminary diagnostic guidelines for SJIA-MAS were proposed in 2005 [15], and new set of classification criteria were proposed in 2016 based on expert consensus and analysis of patient data [16]. These criteria include hyperferritinemia, thrombocytopenia, elevation of aspartate aminotransferase, triglycerides, and hypofibrinogenemia to distinguish SJIA without MAS from patients with clinically overt SJIA-MAS.

The pathologic mechanisms by which MAS develops have not been fully clarified but clinical and translational research has revealed some characteristic findings.

- *Impaired lymphocyte cytotoxicity*. pHLH is caused by genetic defects directly related to lymphocyte cytolytic function, which impair cytotoxicity of cytolytic T lymphocytes (CTL) and NK cells leading to persistent cytokine production and uncontrolled activation of mononuclear phagocytes [17]. In MAS, no single genetic factor has been found but decreased or dysfunction of NK cells during SJIA-MAS has been reported [18–20]. Furthermore, genetic analysis of SJIA and MAS has revealed hypomorphic protein-altering variants in causative genes of pHLH such as *PRF1, MUNC13-4, LYST,* and *STXBP2* in substantial proportion of SJIA MAS patients (20–35.7%) [20–23]. Patients with recurrent MAS were more likely to carry these mutations than patients with SJIA with no history of MAS [22].
- *Persistent activation of tissue macrophages and T cells.* Activation of macrophages has been suggested by histological findings and studies on patient biomarkers. Hemophagocytosis is detected in 60.7% of patients with SJIA-MAS who underwent bone marrow examination [24]. Positivity of CD163 on macrophages and soluble CD163 levels are reported as a sign of macrophage activation and also inflammatory compensation [7, 25, 26]. These activated macrophages are also thought to be a primary source of pro-inflammatory cytokines [27]. Similarly, pHLH is associated with excessive CTL and/or NK cell activation [28]. In MAS, activation of T cells is suggested by high circulating levels of soluble interleukin-2 receptor a (sIL-2Ra) in patients, and the success of T-cell targeting therapies such as cyclosporine A [26, 29]. Recently, increased frequency of CD38high/HLA-DR^+^ activated CD8 T cells has been reported to be a diagnostic marker for childhood HLH [30]. De Matteis et al. reported that a cell population bearing the same surface marker profile was found in SJIA-MAS patients compared to active SJIA, and these cells could be the source of Interferon (IFN)-γ (see below) [31].
- *Hypercytokinemia.* Cytokine storm represents the final common pathway in HLH. Patients with MAS demonstrate enhanced production of pro-inflammatory cytokines such as IL-1, IL-6, IL-18, IFN-γ, and tumor necrosis factor (TNF) or natural antagonists of these cytokines [32, 33]. The most distinguishing feature in SJIA-MAS is extremely high serum IL-18 levels, which are significantly higher than pHLH or other rheumatic diseases [34–37]. Higher IL-18 levels are related to higher risk of MAS, and patients with chronic or polycyclic course show persistently higher serum levels of IL-18 [38]. Furthermore, higher IL-18 is also a risk for SJIA-LD [13]. Detection of free IL-18 caused by imbalance of IL-18 and its endogenous antagonist IL-18BP is also reported [39]. The consequences of this hyper-IL-18 environment are incompletely understood. One study revealed defective phosphorylation of IL-18 receptor beta in SJIA that would be related to impairment of NK cells [40]. While there are currently no clinically available therapeutics targeting IL-18, there is a report of clinical application of recombinant IL-18BP (tadekinig alpha) in one patient with refractory SJIA-MAS [41].
- *Interferon gamma production.* Lastly IFN-γ appears to play a pivotal role in both MAS and other forms of HLH. IFN-γ is mainly produced by NK cells and T cells, and activates monocytes/macrophages into a pro-inflammatory M1 polarization phenotype [42]. Patients in the active phase of SJIA without MAS do not show overproduction of IFN-γ. On the other hand, multiple studies of serum or peripheral blood mononuclear cells suggest IFN-γ production is related to the emergence of MAS [39, 43–45]. Furthermore, some studies described monocytes in SJIA patients exhibiting hyper-responsiveness to IFN-γ, especially in patients treated with IL-1 blockade [46–48]. Recently, gene expression analysis of hemophagocytic bone marrow macrophages in MAS revealed overexpression of TRIM8, which is a positive promotor of IFN-γ pathways [49]. In 2018, the anti-IFN-γ monoclonal antibody emapalumab received FDA approval for treatment of HLH [50], and clinical trials in MAS (NCT05001737) are ongoing with favorable preliminary results [51].

It should be noted that most of these findings regarding MAS were derived from research into patients with SJIA-MAS. It is still unclear whether these are relevant to MAS in other rheumatic diseases such as systemic lupus erythematosus (SLE) [52].

A powerful tool for defining disease pathogenesis is experimental small animal models. Several animal models sharing some clinical features with SJIA and/or MAS have been proposed and studied since 2010s and have been utilized to understand the underlying pathophysiology of these disorders and/or treatment strategies. These models stand in contrast to models of pHLH, which were made using mice with defects in specific causative genes involved in the cytolytic pathway [53], or secondary HLH models when cytokine storm is triggered only after infection with certain viruses [54]. On the contrary, these models for MAS are generally designed to demonstrate hyperinflammation in the setting of persistent systemic immune activation, without specific cytolytic defects or infection. While these models show SJIA and/or MAS-like features, each has unique presentations. The aim of this review is to clarify what is so far known regarding animal models of SJIA and MAS, and what key unanswered questions exist with these experimental systems.

## Animal models for MAS and SJIA

### Repeated TLR-9 stimulation model

**Fig. 1:**
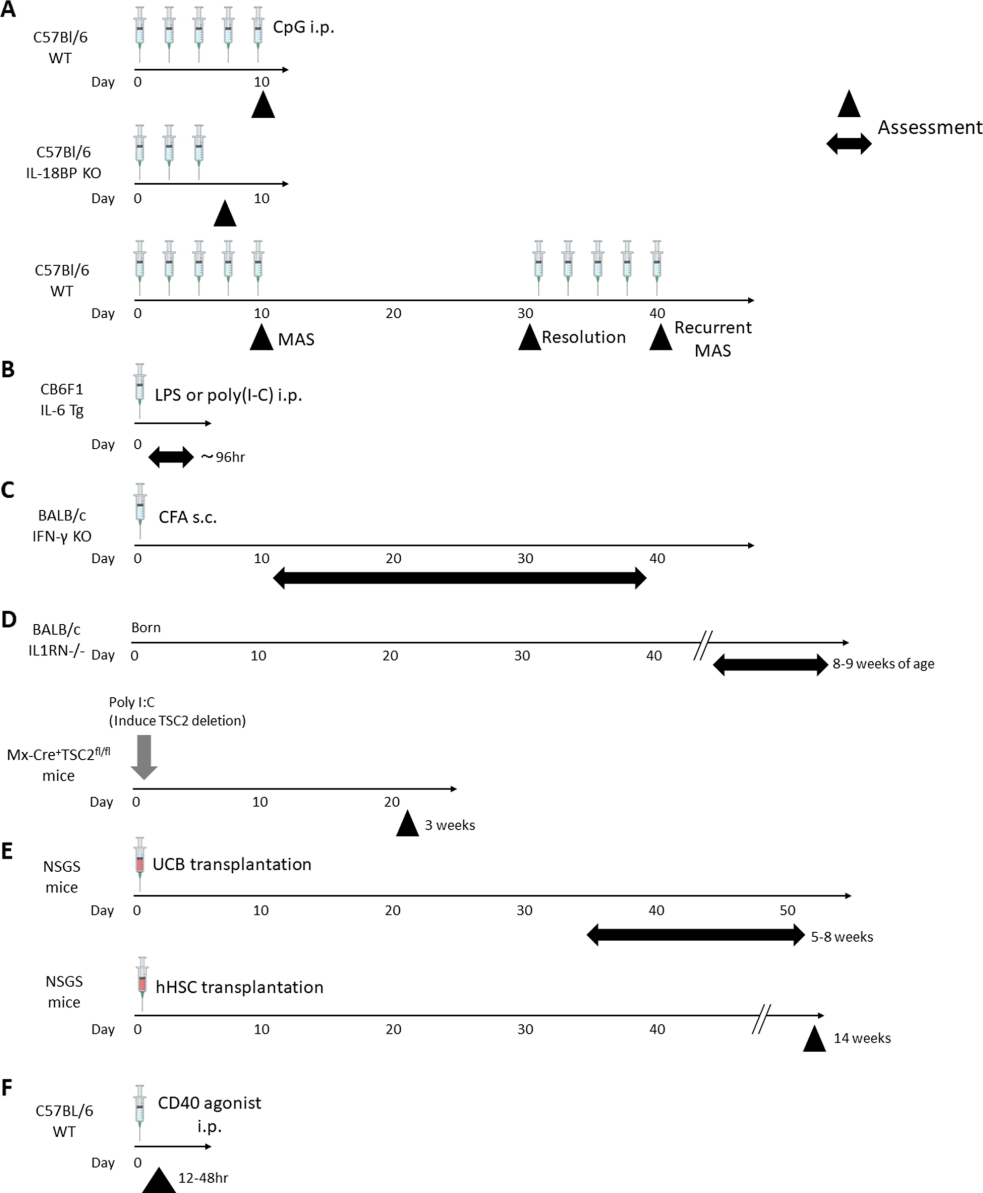
Temporal overview of mouse models of SJIA and MAS. **A** Repeated TLR-9 stimulation model. **B** IL-6 transgenic mice with TLR stimulation model. **C** IFN-γ knockout mice with CFA stimulation model. **D** IL1RN − / − and Tsc2-knock out models. **E** NSGS mice with xenograft models. **F** CD40 stimulated mice model. WT: wild type, i.p.: Intraperitoneal injection, IL-18BP: Interleukin-18-binding protein, KO: knock out, Tg: transgenic, IFN-γ: interferon gamma, CFA: Freund’s complete adjuvant, s.c.: subcutaneous injection, UCB: umbilical cord blood, hHSC: human hematopoietic stem cells

The repeated Toll-like receptor (TLR)-9 stimulation model was first reported in 2011 by Behrens and colleagues [55]. This model was inspired by previous findings that SJIA is associated with altered TLR-related gene expression patterns, especially in patients with higher ferritin levels [33], and that MAS is often triggered by infection. In this model, MAS is induced in wild type (WT) C57Bl/6 mice with intraperitoneal injection of the TLR-9 ligand CpG 5 times in 10 days (Fig. 1A).

Several clinical and laboratory features observed in this model are consistent with MAS, such as splenomegaly, hyperferritinemia, and hypercytokinemia including elevation of IFN-γ, IL-6, and IL-10 (Table 1). Of note, these manifestations are exacerbated by IL-10 blockade, and hemophagocytosis is observed only with IL-10 blockade [56]. In this model, IFN-γ is required for maximal clinical presentation of MAS. Interestingly, IFN-γ KO mice develop some MAS features such as weight loss, hepatitis, thrombocytopenia, and hemophagocytosis which are comparable with WT, but are protected against anemia. CpG suppresses hematopoiesis in bone marrow, and IFN-γ KO leads to erythroid expansion in the spleen, which suggests IFN-γ mediates anemia in part by inhibiting the ability to respond to extramedullary hematopoiesis. IL-12 blockade is also as effective as IFN-γ, suggesting IL-12 may act upstream of IFN-γ. Further studies confirmed that MAS manifestations in this model require both CpG and IFN-γ signaling [57]. Related to IL-10, a recent report suggested heme oxygenase 1 (HO-1) regulates production of IL-10 and that monomethyl fumarate, a drug activating HO-1 production, ameliorates MAS features [58].

**Table 1:** Phenotypic features of animal models for SJIA and MAS

		Repeated TLR-9 stimulation	IL-6 transgenic mice with TLR stimulation	IFN-γ KO/CFA stimulation	IL1RN deficient model	Tsc2 KO model	NSGS mice with xenograft	CD40 stimulation
Clinical manifestations	Fever	nd	nd	-	nd	nd	+	nd
	Arthritis	−	nd	+	+	+	nd	nd
	Skin rash	−	nd	+	nd	nd	nd	nd
	Splenomegaly	+	nd	+	+	+	+	+
	Hepatomegaly	+	nd	−	nd	nd	−	nd
	Lymphadenopathy	nd	nd	+	+	nd	nd	nd
	Weight loss	+	+	+	nd	nd	+	nd
	Fatality	−	+	nd	−	−	+	nd
Laboratory/pathological manifestations	Leukocytopenia	+ (neu↓, Lym↓), myelopoiesis↑	neu↓	− (neu↑, lym↓)	− (neu↑, lym → , inflammatory mono↑)	+ (neu↓, Lym↓)	+	+ (mono↓, lym↓)
	Anemia	+	+	+	+	+	+	nd
	Thrombocytopenia	+	+ (transient)	− (↑)	− (↑)	nd	+	+
	Hemophagocytosis	+ (IL-10 block)	−	+	nd	+	+	+
	Elevated transaminases	nd (+ in IL18BP* − */* − *mouse)	+	nd	nd	nd	nd	+
	Hyperferritinemia	+	+	nd	+	+	nd	+
	sCD25	nd	↑	nd	nd	nd	↑	↑
	Lung manifestation	CD4 predominant interstitial infiltration, IFN-γ related inflammation in alveolar macrophages	nd	Neutrophils/activated macrophages dominant infiltration to subpleural/parenchymal area. Augmented IL-1b, IL-6, G-CSF	nd	nd	nd	nd
	NK cell status	Decreased in spleen, activated	Impaired cytotoxicity	Decreased number, impaired cytotoxicity	nd	nd	nd	Activated
	Liver pathology	Fibrin thrombi, necrosis, lymphohistiocytic infiltrate	Dilated sinusoids and infiltration of CD68 + macrophage	nd	nd	nd	Macrophage infiltration, loss of hepatic parenchyma	Thrombosis, infarction
Cytokines	IL-6	↑	↑	↑	↑(plasma, joint mRNA)	nd	↑	↑
	IL-1β	→	↑	↑ (mRNA levels in LN)	↑ (serum CXCL1, joint mRNA)	nd	nd	↑
	IL-18	↑	↑	↑ (mRNA levels in LN)	nd	nd	nd	nd
	IFN-γ	↑	CXCL9, 10 ↑	−	→	nd	→	↑
	Other cytokines	IL-10, IL-12↑	TNF↑	IL-17↑	TNF↑(joint mRNA), S100A8/A9↑	nd	IL-10, MIP↑	TNF, IL-12, IL-10↑
Interventions	Protective interventions	IFN-γ blockade (anemia, lung), monomethyl fumarate (cytopenia, IFN-γ, hepatosplenomegaly), rapamycin (cytopenia, hyperferritinemia, hepatosplenomegaly, IFN-γ)	IFN-γ blockade (survival, weight loss, ferritin, ALT, hypercytokinemia)	IL-17 blockade (splenomegaly), IL-12/IL-23p40 blockade (weight loss, arthritis, rash, neutrophilia, hepatosplenomegaly)	Anakinra (neutrophilia, monocytosis, thrombocytosis, hyperferritinemia), phagocyte depletion (splenomegaly, arthritis), rapamycin (leukocytosis, monocytosis, anemia, thrombocytosis, splenomegaly, arthritis)	Rapamycin (hemophagocytosis)	Myeloid cell elimination (anemia, splenomegaly), tocilizumab(life span, anemia, inflammatory cytokines)	Macrophage depletion (ALT, inflammatory cytokines)
	Exacerbating interventions	IL-10R blockade, higher free IL-18	nd	IL-10 blockade, NK cell depletion (for WT)	nd	nd	nd	nd
Reference		[55–62]	[63–65]	[66–70]	[71–73]	[72]	[74, 75]	[76]

*nd* Not determined, *neu* Neutrophils, *lym* Lymphocytes, *mono* Monocytes, *IL-18BP* Interleukin-18-binding protein, *sCD25* Soluble CD25, *LN* Lymph node, *ALT* Alanine aminotransferase, *MIP* Macrophage inflammatory protein

In addition to these cytokines, the contribution of IL-18 to the pathogenesis of this model has been assessed. Circulating IL-18 is elevated with repeated CpG treatment [56]. Mice with elevated free IL-18 using transgenic IL-18 or IL-18BP KO have no phenotype at baseline, but demonstrate more severe MAS manifestations upon CpG treatment (Fig. 1A, Table 1) [37, 59]. On the other hand, the clinical presentation of MAS is largely unchanged in mice with NLRC4 gain of function mutations, which in patients lead to hyper-IL-18 and recurrent MAS [77, 78], although notably these animals do not show free IL-18 elevation [37]. Thus, free IL-18 exacerbates the manifestations of MAS, while IL-18BP plays a protective role by blocking free IL-18 in this model [37, 60]. Cellular sources or regulation of IL-18 and IL-18BP production is still not clarified, although it appears to involve both radiosensitive and radioresistant cells [60].

In contrast to mouse models of pHLH, activation of CD8 T cells is not as prominent in the repeated TLR-9 model. MAS features can be obtained in *B2m − / − *mice (lacking CD8 and NKT), *Rag2 − / − *mice (lacking T, B and NKT), and mice after NK cell depletion with antibodies. However, full lack of all types of lymphocytes utilizing *Rag2 − / − Il2rg − / − *mice leads to partial attenuation of cytopenia, splenomegaly, and circulating IFN-γ levels compared with *Rag2 − / − *mice, suggesting a smaller and redundant role for lymphocytes in this model. Indeed, the most notable alternation in this model regards myeloid cells. Weaver et al. showed that TLR-9 and IFN-γ-dependent signals synergically enhance marked extramedullary myelopoiesis, particularly in spleen [57]. It is still unclear precisely which cellular/molecular mechanisms of each cell contribute to MAS pathogenesis in this model.

Several tissue-specific features of the repeated TLR-9 model have also been reported. Studies on liver manifestation in this model revealed that unique CD8 + T infiltrate to liver, which produce IL-10 in a partially IFN-γ-dependent manner. These IL-10 + hepatic CD8 + cells have a signature of activated effector cells with high degree of turnover and responsiveness to liver injury-associated growth factors. These findings indicate that this population is induced by systemic inflammation and then accumulates in the liver with the potential to contribute to tissue injury [61]. Recently, lung pathology in this model was described by Gao et al. [62]. In the acute phase of MAS, histopathological findings exhibit mild but diffuse CD4-predominant interstitial inflammation in the lung. Lung tissue or BAL fluid analysis revealed elevated IFN-γ, its induced chemokines, and IL-18. Alveolar macrophages demonstrate IFN-γ induced transcriptional signatures and pro-inflammatory polarization. In addition, single-cell RNA sequencing of whole lung tissue detected IFN-induced transcriptional changes throughout the lung, as well as MAS-specific macrophage populations with phenotypes suggesting tissue hemophagocytes including apoptotic cell removal and response to IFN-γ. This study also demonstrated both MAS resolution and recurrent MAS with a second round of repeated CpG injection (Fig. 1A). After MAS resolution, most clinical features were resolved and IL-18 remained elevated, as commonly seen with SJIA. In the lung, transcriptional profiles of alveolar macrophages switched towards an anti-inflammatory phenotype after systemic MAS resolution. Notably, recurrent MAS reset this polarization towards a pro-inflammatory state and worsened lung inflammation. A pivotal role of IFN-γ-driven myeloid activation for MAS and LD in this model was suggested by attenuated manifestations in mice with IFN-γ insensitive macrophages. Overall, the pathological and immunological profile of lung inflammation and recurrent MAS exhibited some similarly to that seen in SJIA/MAS-associated LD [62].

Thus far, this model remains the best characterized model of MAS, and closely resembles the pathophysiology of MAS in SJIA, including some features of lung disease seen in patients. It is also notably driven by extensive myeloid activation, with smaller and redundant roles for lymphocytes. However, it should be noted that this model does not exhibit many of the typical features of SJIA, such as apparent arthritis, rashes, or marked neutrophil expansion, supporting this system as a more “pure” model of MAS.

### IL-6 transgenic mice with TLR stimulation model

IL-6 is one of the pivotal cytokines in the pathogenesis of SJIA, as prominent IL-6 production has been shown as well as the clinical efficacy of the anti-IL-6 receptor antibody tocilizumab (TCZ) [79, 80]. Strippoli et al. [63] hypothesized that cooperation of IL-6 and TLR elicited signals could amplify inflammatory responses, as seen in MAS triggered by infections in SJIA. Mice used in this model are IL-6-transgenic (IL-6TG) and demonstrate high circulating levels of human IL-6 beginning at birth, with growth defects but no sign of chronic tissue inflammation [81]. In contrast, IL-6TG mice treated with a single administration of LPS reveal decreased survival with increased production of pro-inflammatory cytokines such as TNF, IL-1β, and IL-18 compared to WT mice (Fig. 1B). Clinically, significant cytopenias, increased soluble CD25 (sCD25), ferritin, and LDH were observed compared to WT (Table 1). Ex vivo experiments revealed that intracellular IL-6 signaling in macrophages from IL-6TG mice is increased and more markedly increased by LPS [63].

Cifaldi et al. focused on NK cell cytotoxicity in this model as one key pathogenic mechanism of MAS. Splenic NK cells from IL-6TG mice treated with poly(I-C) showed impaired cytotoxicity, and this impairment was caused by reduced expression of perforin and granzyme B without altered granule exocytosis [64]. Prencipe et al. assessed whether IFN-γ is involved in pathogenesis of this model [65]. IFN-γ signature, including circulating levels, gene expression of IFN-γ and inducible chemokines in liver/spleen, was upregulated. IFN-γ neutralization improved survival and clinical manifestations such as weight loss, elevation of plasma ferritin, alanine aminotransferase, and hypofibrinogenemia along with downmodulation of circulating pro-inflammatory cytokines (IL-1β, IL-6, and TNF) [65].

Overall, chronic IL-6 production appears to amplify MAS-like features after LPS administration. However, important information including clinical features such as arthritis, hepatosplenomegaly, and hemophagocytosis has not been described despite this model being first proposed to demonstrate SJIA-MAS. Furthermore, tissue-specific features of inflammation, particularly in the lungs, are expected to be reported.

### IFN-γ knockout / CFA stimulation model

In 2014, Avau and colleagues reported an animal model of SJIA wherein IFN-γ-knockout (IFN-γ-KO) mice were administered Freund’s complete adjuvant (CFA) [66]. CFA contains heat killed mycobacterium which triggers innate and adaptive immunity [82], and IFN-γ-KO mice on the BALB/c background are used in animal models of other autoimmune diseases induced with CFA [83, 84]. In this model, CFA treatment induced weight loss, splenomegaly, lymphadenopathy, thrombocytosis, neutrophilia, anemia, and arthritis with histological evidence of synovitis. These features were more severe after CFA treatment in IFN-γ-KO mice, and only IFN-γ-KO mice showed skin rashes (Fig. 1C, Table 1). IFN-γ-KO mice also showed anemia with increased immature erythroid and myeloid cells in blood, and hemophagocytosis in multiple organs. Hypercytokinemia was seen including elevation of serum IL-6, IL-1β, IL-18, and IL-17. Anti-IL-17 and anti-IL-12/IL-23p40 treatment reversed SJIA-like features such as weight loss, splenomegaly, and neutrophilia. CFA-challenged IFN-γ-KO mice showed increased expression of IL-17 in CD4 + T cells and γ/δ T cells, which suggested both adaptive and innate lymphocytes are involved in IL-17 production [66].

Imbrechts et al. assessed the involvement of IL-10 in this model [67]. Ex vivo experiments showed IL-10 production in splenocytes and lymph node cells was decreased in CFA-challenged IFN-γ-KO mice compared to WT. IL-10 blockade resulted in more pronounced SJIA characteristics such as weight loss, splenomegaly, thrombocytosis, and neutrophilia in CFA-challenged WT mice. CFA-challenged WT mice with IL-10 blockade showed increased osteoclast precursor cells in spleen, as seen in IFN-γ-KO mice, which they suggested reflecting the early phase of arthritis [67].

NK cell function in this model was assessed by Vandenhaute et al. NK cells in CFA-treated IFN-γ-KO mice were reduced in number and showed increased proportion of activation markers and impaired cytotoxicity with reduced expression of perforin and granzyme B compared to WT, similar to that seen in patients with SJIA. Both NK cell depletion and blockade of NK cell activation receptors (NKG2D) lead to worsened CFA-induced inflammation in WT mice. Furthermore, degranulation of NK cells to autologous activated immune cells was most prominent towards monocytes, and this function was suppressed by IFN-γ-KO and NKG2D blockade. Overall, these findings suggest this model may be helpful to study the role of NK cells in regulating activated immune cells, predominantly monocytes, in SJIA and MAS [68].

Recently, Malengier-Devlies et al. described the role of G-CSF in this model. G-CSF induces neutrophilia, extramedullary myelopoiesis, arthritis, and hypercytokinemia. Characterization of neutrophils using surface markers and single-cell RNA sequencing revealed that G-CSF induced immature neutrophils and myeloid derived suppressor-like cells. Intriguingly, manifestations such as extramedullary myelopoiesis and arthritis were ameliorated by G-CSF blockade but weight loss and elevation of plasma cytokine levels such as IL-6, IL-17, and TNF-α were worsened [69]. Overall, G-CSF and neutrophils appear to play both pathogenic and protective roles in this model.

Finally, features of lung disease have also been reported in this model [70]. Here, lungs exhibited subpleural and parenchymal cellular infiltrates consisting mainly of neutrophils and activated macrophages, with augmented expression of IL-1β, IL-6, and G-CSF. Notably however, both the pathological finding of neutrophil activation and that these manifestations are worse in IFN-γ-KO mice, is not consistent with emerging data regarding SJIA-LD [13].

Overall, this model is clinically much closer to SJIA than other models, given significant neutrophil activation and prominent findings of rash and histological arthritis, which is one of the most significant features for patients especially with a chronic SJIA course. In contrast, this model may represent a less relevant model for MAS as this system requires IFN-γ-KO, given the established role for IFN-γ in MAS. Hemophagocytosis and NK cell dysfunction observed in this model may represent the “occult MAS” in SJIA patients.

### IL1RN-deficient model and Tsc2-knock out model

In 2000, Il1RN* − */* − *mice on a BALB/cA background were reported to spontaneously develop an inflammatory polyarthritis with elevated immunoglobulin and autoantibodies, which was described as a rheumatoid arthritis-like model [71]. Although this system is most technically a model of deficiency of IL-1 receptor antagonist (DIRA), caused by biallelic loss-of-function mutations in the *Il1RN* gene [85, 86], it clinically resembles SJIA. Of note, *IL1RN* variants are related to disease susceptibility of SJIA and cause lower *IL1RN* gene expression and reduced levels of the natural IL-1 receptor antagonist [87].

Recently, Huang et al. reframed the Il1RN-deficient model as reflecting SJIA-like symptoms, including leukocytosis (neutrophilia, increase of inflammatory monocytes), thrombocytosis, anemia, and splenomegaly along with arthritis (Table 1). Single-cell transcriptomic assessment of this model showed the upregulated expression of mechanistic target of rapamycin complex 1 (mTORC1), especially in monocytes. Furthermore, mTORC1 activation by induced deletion of Tsc2, a negative regulator of mTORC1, leads to spontaneous arthritis along with MAS-like features such as splenomegaly, leukocytopenia, anemia, hyperferritinemia, and hemophagocytosis (Fig. 1D, Table 1). Rapamycin, an inhibitor of mTORC1, ameliorates most of these symptoms in the IL1RN deficiency model and fully prevented hemophagocytosis in Tsc2 KO animals. Interestingly, the activation of mTOR pathway is also seen in the repeated TLR-9 model, and rapamycin significantly ameliorates MAS symptoms (cytopenia, hyperferritinemia, hepatosplenomegaly, and plasma IFN-γ levels) [72]. The precise molecular mechanisms of how mTOR pathway could affect the pathophysiology of SJIA and MAS have not been clarified, but these overall results suggest that mTOR pathway, specifically in monocytes, could be a candidate treatment target.

Interestingly, in 2016, Geven et al. utilized the IL1RN* − */* − *mouse model to study the usefulness of S100A8/A9 as a biomarker for inflammatory arthritis. This study showed that serum levels of S100A8/A9 were significantly correlated with clinical and pathological severity of arthritis. Furthermore, inflamed synovium was detected as a source of this molecule [73]. Notably, S100 proteins including S100A8/A9 have been reported as useful biomarkers for diagnosis, disease activity, and prognosis in SJIA [88, 89].

### NSGS mice with xenograft model

NSG mice (NOD/SCID-IL-2Rgnull) have a complete lack of lymphoid cells as well as defects in innate immunity. This strain enables efficient engraftment of human immune cells, but establishment of myeloid cells is limited [90]. NSGS mice were generated in order to solve this problem, adding transgenic expression of human stem cell factor (SCF), IL-3 and GM-CSF. As a result, more efficient engraftment of human myeloid cells was achieved [91]. With these NSGS mice, MAS-like phenomenon has been reported upon transplantation of some human-derived cells.

Wunderlich et al. first reported NSGS mice engrafted with human umbilical cord blood (UCB) developed MAS-like features, such as decreased survival, fever, pancytopenia, splenomegaly, and elevation of sCD25. Plasma cytokine levels such as MIP1a,b, IL-1Ra, IL-6, and IL-10 were elevated but IFN-γ and TNF were not (Fig. 1E, Table 1). In addition, tocilizumab was effective to prevent clinical progression and prolong lifespan. Of note, NSG mice or NSGS mice with wild type IL-2Rg bearing normal endogenous NK cells did not develop MAS-like features. Furthermore, lymphocyte-suppressive treatments did not prevent MAS presentation but elimination of myeloid cells with gemtuzumab ozogamicin completely reversed clinical symptoms [74]. These findings suggest MAS features in this model are driven mainly by the myeloid lineage, while NK cells play a protective role.

In addition, MAS-like features were triggered in NSGS mice by transplantation of human hematopoietic stem cells (hHSC) (Fig. 1E) [75]. These mice exhibited decreased survival, lethargy, BW loss, splenomegaly, small and irregular liver, cardiomegaly, thrombocytopenia, and anemia (Table 1). Pathologically, mice showed infiltration of activated macrophages which are chimeric, including hemophagocytosis in multiple organs and activation of the inflammasome.

While these two models do show MAS-like clinical features, given the complex immune interactions present in xenograft models, it is unclear how much can be applied from these models to understand pathophysiology of human diseases like SJIA and MAS. However, they may be useful models to understand the pathologic consequences of unconstrained pro-inflammatory myeloid cell activation in tissue.

### CD40 stimulated mice model

Finally, Ingoglia et al. described a murine model driven by stimulation of CD40, a costimulatory receptor expressed on antigen-presenting cells, which leads to an activation of innate and adaptive immunity [76]. Immediately after injection of CD40 agonistic antibody, wild type mice developed liver damage, splenomegaly with B cell activation, elevation of sCD25 and ferritin, coagulopathy, cytopenia, and hypercytokinemia (Fig. 1F, Table 1). The authors concluded that macrophages are the key player in this model using conditional knock out of CD40 in macrophages or macrophage depletion with clodronate liposomes. Other types of leukocytes are also activated by CD40, but depletion of these cells did not achieve complete reversal of inflammatory features [76]. Similar to the NSGS systems, the phenotype induced in this model is quite literally “macrophage activation.” However, the relationship with the pathophysiology of MAS in a background of rheumatic diseases is unclear.

## What remains to be clarified in the future

The underlying causes of SJIA and MAS remain unknown. However, they in general are not felt to be due to a single genetic lesion as seen in pHLH. As such, while animal models are critical tools to study disease pathogenesis and provide pre-clinical data for novel treatment approaches, an ideal animal model for SJIA MAS remains elusive. However, as described above, several proposed mouse models exhibit clinical presentations that partially overlap with SJIA and/or MAS, although each has own features, benefits, and limitations.

Examining proposed animal models and human SJIA and MAS, there are some differences which cannot be ignored. First, SJIA patients, especially those complicated by MAS, show extremely high IL-18 (often > 100,000 pg/ml), and this elevation persists chronically [34, 35]. While some of these mouse models show elevated IL-18 at the onset of MAS-like disease or even in recovery phase [62], these are orders of magnitude less than seen in SJIA and MAS. Notably, several approaches have attempted to augment IL-18, particularly in the repeated TLR-9 model, with mixed results [37, 59, 60]. To further explore these approaches, comprehensive gene expression profiles by Weiss et al. shows that expressional distribution of IL-18 and IL-1β has distinct pattern in mice. Of note, *IL1b* is expressed predominantly in neutrophils together with inflammasome molecules, whereas *IL18* and *Nlrc4* expression is seen predominantly in epithelial cells [37]. This strategy and finding could be one clue to reveal how extremely high IL-18 elevation in SJIA and MAS could be modeled. Second, arthritis is not observed in the majority of these models, while many of those that do develop arthritis such as the CFA stimulation model show less classic MAS features. Arthritis is included in currently used classification criteria of SJIA [9, 92], although not universally seen, but is not an essential factor in a model of MAS. An ideal animal model for SJIA-MAS would thus have chronically elevated IL-18 and SJIA-like clinical features, and develop complicating MAS after some trigger.

Another central role for animal models is recognized as useful tools to study novel treatment options. Even in the present era of biologics blocking IL-1 or IL-6, there are still patients suffering from refractory courses of SJIA and MAS [93]. When utilizing animal models of SJIA or MAS, we need to choose a suitable model considering what we want to evaluate and the specific clinical and immunological features of each model. Looking at clinical manifestations, for instance, the CFA stimulation model and IL1RN deficiency/Tsc2 KO models may be more suitable for studying chronic and arthritic subtypes of SJIA, while the repeated TLR-9 model may be preferable for studying lung inflammation. In terms of pathophysiology, the IFN-γ axis is involved in both repeated TLR-9 and IL-6TG model, while CFA stimulation model is actually worsened by IFN-γ-KO but shows a key role for IL-17. IL-18 involvement and particularly free IL-18 has been assessed only in repeated TLR-9 model. On the other hand, impaired NK cell cytotoxicity is seen in IL-6TG and CFA stimulation models, while NK cells are largely dispensable in the repeated TLR-9 model. Corresponding to these findings, efficacy of several intervention and treatments has been shown in some systems. IFN-γ blockade has been shown to be effective in the repeated TLR-9 model, while monomethyl fumarate, which upregulates IL-10 production via HO-1, ameliorates cytopenia and hepatitis. IL-6 blockade has not been well studied in MAS animal models, but the NSGS model showed partial response to tocilizumab. Interestingly, there is very limited reports of efficacy of recombinant IL-1Ra or IL-1β inhibitors in animal models (except for IL1RN deficiency model), which are both approved biologics in SJIA. With the results from the recent study on IL1RN deficiency and Tsc2 KO model, rapamycin targeting mTORC1 may be an additional candidate agent.

Finally, chronic lung disease in the setting of SJIA and MAS has been increasingly recognized in the past decade, in parallel with widespread use of anti-cytokine (IL-1 and IL-6) biologics [12–14]. The underlying causes of SJIA-LD have not been fully clarified yet. So far, several pathogenic mechanisms have been proposed including delayed drug reaction related to HLA-DRB1*15:XX, effects of the altered cytokine milieu on alveolar macrophage functions, and the “cytokine plasticity hypothesis” of skewed T cell responses [13, 94, 95]. Due to these concerns, there are urgent unmet needs for new approaches to therapy for SJIA and MAS, including pre-clinical studies with animal models. One potential approach is biologic agents targeting cytokines other than IL-1 or IL-6. Prior animal studies revealed the repeated TLR-9 model requires IFN-γ for full manifestation of MAS or lung findings; IFN-γ blockade also ameliorates symptoms in IL-6TG mouse too. Currently, emapalumab (anti-IFN-γ monoclonal antibody) is in clinical trials for refractory MAS with favorable interim results [51]. Another approach is blocking IL-18. Animal studies also revealed a protective role of IL-18BP in repeated TLR-9 model. One case report of effectiveness of tadekinig alpha (recombinant human IL-18BP) for SJIA has been reported [41], and a small clinical trial for AOSD showing promise [96]. Second, a multiple cytokine blockade strategy is emerging. Double blockade of IL-1 and IL-18 was previously reported effective to reduce severity and mortality in a mouse sepsis model [97]. In human, the effectiveness of MAS825, a bispecific monoclonal antibody targeting IL-1β and IL-18, has been reported in a single SJIA-LD patient [98]. Lastly, both conventional DMARDs such as calcineurin inhibitors (CNI), as well as JAK inhibitors (Jakinibs), are considered options for treatment of MAS. CNI has been used in treatment of MAS for decades [99], while case reports suggested the effectiveness of Jakinibs in SJIA-LD [100] and refractory SJIA/AOSD [101]. Thus, animal models represent important tools to address these candidate options and identify which patients could be most benefitted.

## Conclusion

On a broad level, as the pathophysiology of SJIA/MAS is not completely clarified, it may be impossible to make a “perfect” animal model of these conditions. However, accumulating evidence from multiple animal models shows that some aspects of SJIA and MAS pathogenesis can be effectively replicated with experimental animal systems. Combining translational data in patients with thoughtful use of animal models for distinct clinical, immunologic, and tissue-specific studies will continue to improve our understanding of SJIA and MAS.

## Data Availability

Not applicable.

## Abbreviations

MAS: Macrophage activation syndrome
HLH: Hemophagocytic lymphohistiocytosis
pHLH: Primary hemophagocytic lymphohistiocytosis
SJIA: Systemic juvenile idiopathic arthritis
AOSD: Adult-onset Still’s disease
LD: Lung disease
CTL: Cytotoxic T lymphocyte
NK cells: Natural killer cells
IFN: Interferon
TNF: Tumor necrosis factor
IL: Interleukin
HO: Heme oxygenase
IL-18BP: Interleukin-18-binding protein
SLE: Systemic lupus erythematosus
TLR: Toll-like receptor
WT: Wild type
TCZ: Tocilizumab
sCD25: Soluble CD25
KO: Knock out
CFA: Freund’s complete adjuvant
DIRA: Deficiency of IL-1 receptor antagonist
CNI: Calcineurin inhibitors
Jakinibs: Janus kinase inhibitors
